# Acute Stress Disorder among Tunisian Population in the Palestine-Israel War

**DOI:** 10.1192/j.eurpsy.2024.1720

**Published:** 2024-08-27

**Authors:** N. Messedi, M. Sehli, A. samet, I. Chaari, F. charfeddine, L. Aribi, J. Aloulou

**Affiliations:** ^1^Psychiatry B, Hedi Chaker University Hospital of Sfax, sfax, Tunisia

## Abstract

**Introduction:**

The Gaza-Israel conflict has far-reaching consequences that extend beyond the immediate geographic confines of the conflict zone.This war certainly has repercussions on people who follow it via the media.

**Objectives:**

To study the prevalence of acute stress disorder among Tunisian people and determinate the factors associated to it.

**Methods:**

It was a cross-sectional, descriptive and analytical study, conducted among Tunisians. Data were collected during October and November 2023, through an anonymous online questionnaire, spread throughout social media (Facebook/Instagram), using the Google Forms® platform.

We used the the National stressful Events survey acute Stress Disorder Short scale (NSESSS) to assess the severity symptoms of acute stress disorder .

The National Stressful Events Survey Acute Stress Disorder Short Scale (NSESSS) is a 7-item patient assessment measures that assesses the severity symptoms of acute stress disorder in individuals age 18 and older following an extremely stressful event or experience.

**Results:**

A total of 1091 participants completed the questionnaire. The participants had a mean age of 32.7 ± 9.8 years. More females (77.7%) than males (22.3%) participated in the study with a sex ratio (F/M) = 3.5. They were divorced in 2.1% .A history of psychiatric follow-up was found in 19,5% of case.

Results demonstrated that 100% of the respondents closely monitored the war, primarily relying on social media (98.6%) as their primary source of information.

According to the NSESSS ,83.4% of the participants had an acute stress disorder. The breakdown of acute stress disorder severity indicated that 29.7% experienced mild symptoms, 27.5% moderate, 21.6% severe, and 4.6% extreme symptoms.

The factors associated with high score of NSESSS were: female sex ( p=0.000), the divorced people (p=0.001)and previous history of psychiatric follow-up (p=0.000)

**Conclusions:**

These findings indicate a substantial impact of the Palestine-Israel conflict on the mental well-being of the Tunisian population, as evidenced by high rates of acute stress disorder.

Understanding the heightened prevalence of acute stress disorder among different demographic groups following such international conflicts is crucial for developing tailored interventions to support the mental health and well-being of affected individuals.

**Disclosure of Interest:**

None Declared

